# Signatures of the autonomic nervous system and the heart’s pacemaker cells in canine electrocardiograms and their applications to humans

**DOI:** 10.1038/s41598-020-66709-z

**Published:** 2020-06-19

**Authors:** Aviv A. Rosenberg, Ido Weiser-Bitoun, George E. Billman, Yael Yaniv

**Affiliations:** 1 Computer Science Faculty, Technion-IIT, Haifa, Israel 32000; 2 Biomedical Engineering Faculty, Technion-IIT, Haifa, Israel 32000; 3 Department of Physiology and Cell Biology, The Ohio State University, Columbus, Ohio USA

**Keywords:** Physiology, Cardiology, Diseases

## Abstract

Heart rate and heart rate variability (HRV) are mainly determined by the autonomic nervous system (ANS), which interacts with receptors on the sinoatrial node (SAN; the heart’s primary pacemaker), and by the “coupled-clock” system within the SAN cells. HRV changes are associated with cardiac diseases. However, the relative contributions of the ANS and SAN to HRV are not clear, impeding effective treatment. To discern the SAN’s contribution, we performed HRV analysis on canine electrocardiograms containing basal and ANS-blockade segments. We also analyzed human electrocardiograms of atrial fibrillation and heart failure patients, as well as healthy aged subjects. Finally, we used a mathematical model to simulate HRV under decreased “coupled-clock” regulation. We found that (a) in canines, the SAN and ANS contribute mainly to long- and short-term HRV, respectively; (b) there is evidence suggesting a similar relative SAN contribution in humans; (c) SAN features can be calculated from beat-intervals obtained *in-vivo*, without intervention; (d) ANS contribution can be modeled by sines embedded in white noise; (e) HRV changes associated with cardiac diseases and aging can be interpreted as deterioration of both SAN and ANS; and (f) SAN clock-coupling can be estimated from changes in HRV. This may enable future non-invasive diagnostic applications.

## Introduction

Heart rate variability (HRV) refers to the ever-present variations in the time-intervals between consecutive heartbeats. It is quantified by analyzing beat-interval signals, which consist of these inter-beat time intervals. HRV has been widely documented in mammals, including humans^1,2^. In recent years, changes in heart rate variability have been associated with cardiac diseases and timing of arrhythmogenic events^3,4^. Understanding the mechanisms that contribute to changes in HRV may allow us to target the underlying mechanisms behind cardiac diseases and is the first step for effective treatment.

The average heart rate and its variability are mainly determined by two mechanisms. The first is the autonomic nervous system (ANS)^5,6^, which interacts with receptors on the membrane of sinoatrial node (SAN) cells. The SAN is a region of specialized cardiomyocytes which are capable of spontaneously beating and constitute the heart’s principal pace-making region. The ANS has two signaling pathways affecting the SAN cells: sympathetic stimulation, which increases the heart rate, and parasympathetic stimulation which decreases it. The second mechanism influencing HRV is the “coupled-clock” system within the SAN cells^7^. This system is comprised of two intrinsic intercellular mechanisms (“clocks”), which interact with each other and are able to oscillate even without neural input^2,8^. Although the contribution of both systems to HRV has been acknowledged^9^, their relative contribution to changes in HRV is not known. Understanding the individual contributions of the ANS and SAN to HRV changes may assist in identifying the specific deteriorated system underlying cardiac diseases.

We hypothesized that specific features, which represent the “signatures” of the ANS and SAN mechanisms in beat interval signals, can be obtained from beat-interval signals derived from standard electrocardiogram (ECG) recordings. To assess these individual contributions, we analyzed canine electrocardiograms containing segments of pharmacological denervation, also known as neural double blockade. This is a method of temporarily blocking the ANS by administering a combination of drugs, atropine and propranolol, which respectively block cholinergic and adrenergic receptors. Pharmacological denervation was previously applied to humans and other mammals^9–11^. Although various HRV analysis algorithms were applied to data obtained during denervation, a specific SAN “signature” was not sought. Moreover, some authors attribute all HRV changes to ANS regulation alone, altogether disregarding the effect of the SAN^12^.

We analyzed the differences in HRV between basal and ANS blockade conditions in the canine data to reveal the SAN and ANS contributions and defined a set of features which we designate as the “signatures” of these systems. Furthermore, we demonstrated that these features can be calculated from beat-interval time series obtained *in-vivo*, without any pharmacological or invasive intervention. To identify these features in humans and to determine the effect of pathology and aging on them, we then analyzed human electrocardiograms of young healthy subjects, patients with atrial fibrillation or congestive heart failure, and of healthy, aged subjects. Finally, we used a mathematical model to establish that the degree of clock-coupling in the SAN can be estimated by observing changes in frequency content and nonlinear HRV metrics.

## Results

### Heart rate variability analysis of basal and ANS-blockade segments

We acquired 27 canine ECG records from Billman *et al*.^11^, each containing at least 5 minutes of both basal (denoted as *BSL*) and ANS blockade segments (denoted as *ABK*) and analyzed them with a wide range of HRV metrics. The analysis was performed with the PhysioZoo^13^ platform, which also adapts the HRV metrics for canines and other mammals^14^. We used various HRV metrics that can be broadly categorized into time-domain, frequency-domain (spectral), and nonlinear methods, which quantify physiological complexity^12^.

**Figure 1:**
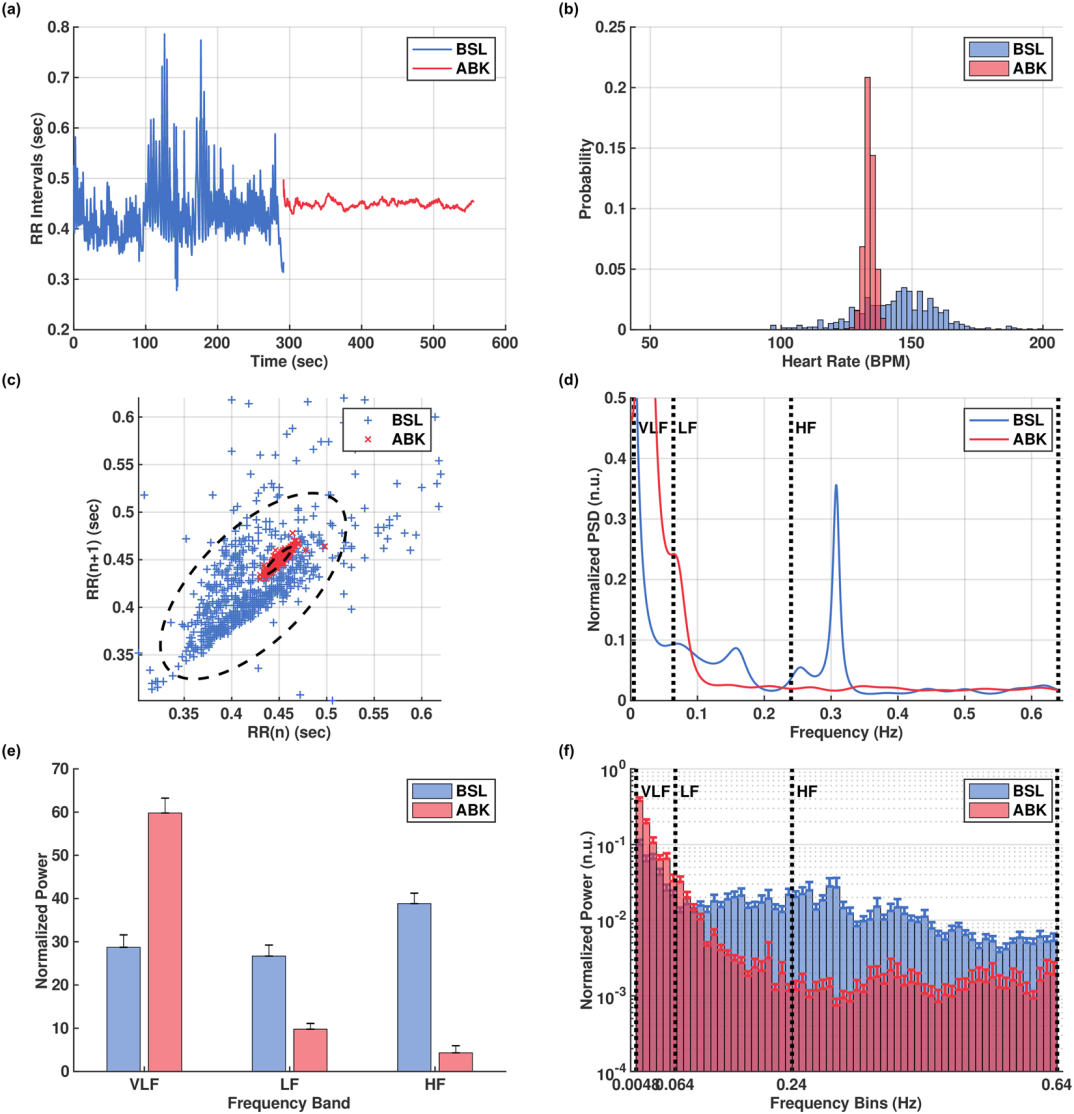
Canine beat intervals in the basal (BSL) and autonomic nervous blockade (ABK) states. Representative examples from one canine, showing visually reduced variability after ABK: (**a**) beat intervals; (**b**) beat interval distribution; (**c**) Poincaré plot; (**d**) normalized power spectral density, showing also the elimination of the ANS-related peaks (baroreceptor, 0.15 Hz and respiratory, 0.3 Hz) after ABK. Averaged values of: (**e**) normalized power in the very low frequency (VLF), low frequency (LF) and high frequency (HF) bands, showing a roughly twofold increase in normalized VLF power after ABK; (**f**) histogram of normalized power, showing the shift of spectral power density towards the VLF band. Vertical bars represent standard error. Figures rendered with Matlab^41^.

The average beat interval decreased after ANS blockade from 518 ± 19 to 460 ± 11 ms (Fig. 1a). Thus, ANS activity leads to a longer average beating interval duration compared to SAN activity alone. Time-domain HRV analysis revealed significant reductions in all metrics following ANS blockade (Table 1). Figure 1b,c demonstrate the reduction of variability after ANS blockade with a representative example. The SAN alone generates beat intervals that change slowly with very small variations from beat to beat compared to the basal case (Fig. 1a–c). The most prominent reductions in time domain metrics were in the pNN32, RMSSD and SD1 measures (Table 1). These metrics all correspond to short term, or beat-to-beat, changes in the heart rate. Therefore, these results suggest that the ANS contributes mostly to the short-term variability.

**Table 1:** Heart rate variability (HRV) analysis results for the canine dataset under the basal (BSL) and autonomic blockade (ABK) states.

		BSL (*N* = 24)	ABK (*N* = 27)	p-value
**Time Domain**				
AVNN	(ms)	516.1 ± 17.9	460.3 ± 11.3*	0.01
SDNN	(ms)	73.4 ± 6.3	21.6 ± 2.1*	0.00
RMSSD	(ms)	74.4 ± 9.7	9.5 ± 1.1*	0.00
pNN32	(%)	46.9 ± 3.8	1.1 ± 0.3*	0.00
SD1	(ms)	52.7 ± 6.9	6.7 ± 0.8*	0.00
SD2	(ms)	88.0 ± 6.3	29.0 ± 3.0*	0.00
**Frequency Domain**				
HF Power	(ms^2^)	1391.9 ± 315.2	23.3 ± 3.8*	0.00
HF Norm.	(n.u.)	39.5 ± 2.4	12.6 ± 1.9*	0.00
HF Peak	(Hz)	0.4 ± 0.0	0.4 ± 0.0*	0.03
LF Power	(ms^2^)	726.6 ± 117.5	26.0 ± 4.2*	0.00
LF Norm.	(n.u.)	26.2 ± 2.1	13.3 ± 1.4*	0.00
LF Peak	(Hz)	0.2 ± 0.0	0.2 ± 0.0	0.70
VLF Power	(ms^2^)	783.7 ± 139.9	138.7 ± 25.1*	0.00
VLF Norm.	(n.u.)	29.1 ± 2.4	52.8 ± 2.6*	0.00
LF/HF	(n.u.)	0.7 ± 0.1	2.2 ± 0.5*	0.01
Tot. Power	(ms^2^)	3057.2 ± 557.7	270.3 ± 52.2*	0.00
Ex-A. Norm.	(n.u.)	7.2 ± 0.8	8.8 ± 0.9	0.18
Ex-B. Norm.	(n.u.)	4.1 ± 0.5	4.2 ± 0.5	0.92
**Nonlinear**				
*β*	(n.u.)	−0.7 ± 0.1	−1.2 ± 0.1*	0.00
*α*_1_	(n.u.)	0.8 ± 0.0	1.3 ± 0.1*	0.00
*α*_2_	(n.u.)	0.8 ± 0.0	1.3 ± 0.0*	0.00
SampEn	(n.u.)	1.4 ± 0.1	0.5 ± 0.1*	0.00
MSE5	(n.u.)	1.6 ± 0.1	0.8 ± 0.1*	0.00
MSE10	(n.u.)	1.4 ± 0.1	1.2 ± 0.1	0.23
MSE15	(n.u.)	1.4 ± 0.1	1.3 ± 0.1	0.33
MSE20	(n.u.)	1.3 ± 0.1	1.2 ± 0.1	0.50

See e.g.^12,13^ for definition of each HRV metric. Reported frequency domain metrics are based on autoregressive (AR) modeling. Ex-A and Ex-B are frequency bands which were found to be invariant under autonomic blockade, corresponding to 0.0450–0.0750 Hz and 0.0750–0.1050 Hz respectively. Results reported as Mean ± SE; *N* is the number of 5-minute segments analyzed from all records; an asterisk denotes *p* < 0.01 in paired t-test.

Examining the effects of ANS blockade in the frequency domain sheds further light on the relative contribution of the SAN to HRV. Results are reported in Table 1. Figure 1d shows a representative example of power spectral density (PSD) before and after ANS blockade. Power in the LF and HF bands is usually attributed to regulatory autonomic processes such as the baroreceptor reflex and respiratory sinus arrhythmia (RSA)^4,15^. Our results show that the characteristic respiratory peak in the high frequency (HF) band and baroreceptor-reflex peak in the low frequency (LF) band are completely abolished after ANS blockade. Looking at the normalized power per band before and after ANS blockade, we found a significant reduction in both the LF and HF bands, while a roughly twofold increase was observed in the VLF band (Fig. 1e). In terms of absolute power, a 91% reduction was observed over the entire frequency spectrum. This suggests that the ANS adds substantial broadband power. Such an addition manifests as added “noise” in the time domain, which corresponds to increased short-term, beat-to-beat variability as shown by the time-domain metrics. These results show that the primary spectral contribution of the SAN is in the VLF band. Moreover, the LF and HF band power is determined predominantly by the ANS. Another interesting finding is so-called “invariant” regions in the normalized PSD (Fig. 1f; Table 1 Ex-A/B Norm.). In these regions, found roughly at 0.035 Hz and 0.075 Hz in the canine data, the normalized spectral power shows no significant change after ANS blockade.

**Figure 2:**
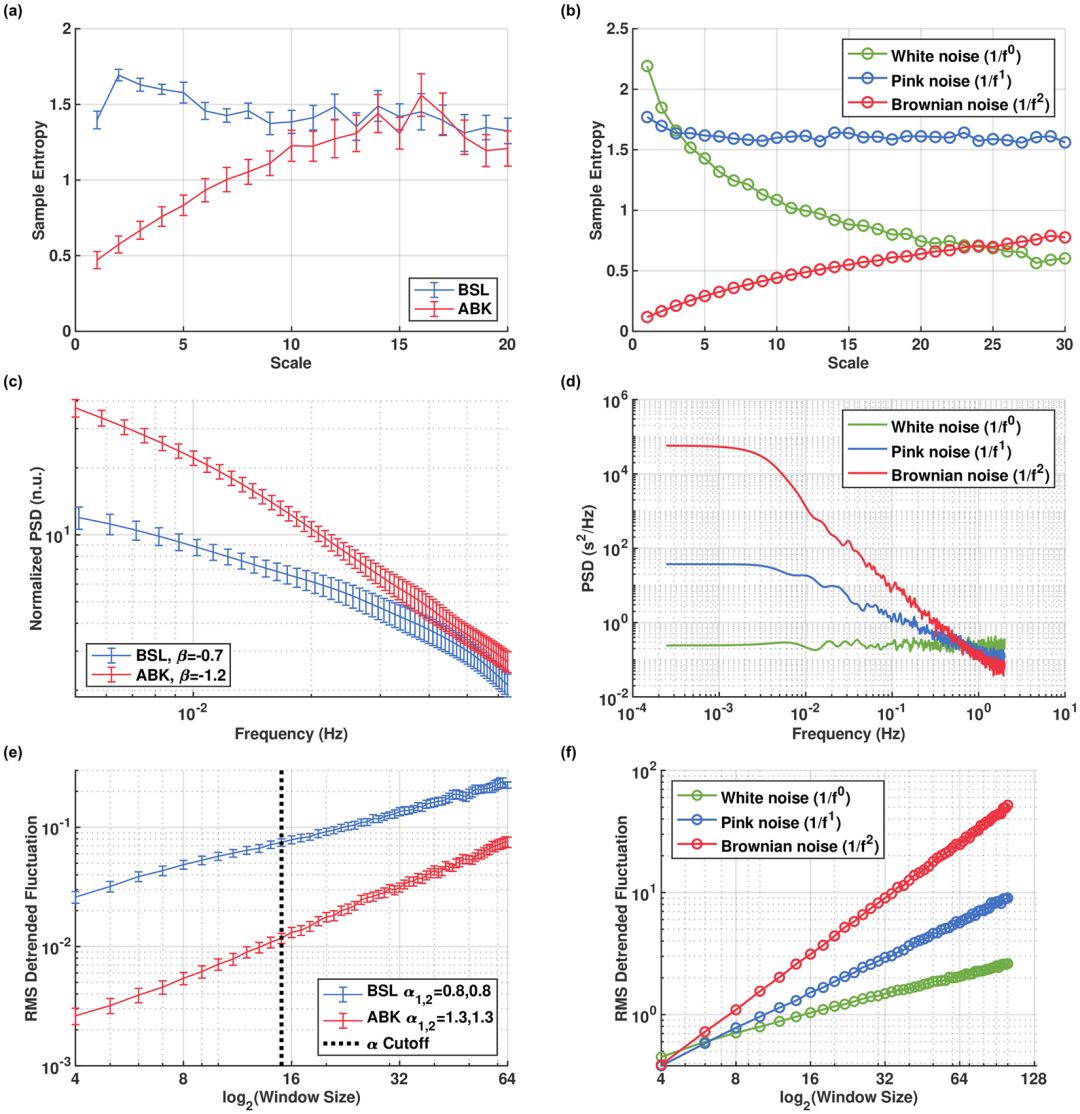
Fractal and complexity metrics of beat intervals in basal (BSL) and autonomic nervous blockade (ABK) states of the canine data, compared to ideal white, pink and Brownian noise processes. (**a**) Average multiscale entropy (MSE); (**b**) MSE of ideal noise, showing a monotonically decrease for while noise, high complexity on all scales for pink noise and a monotonic increase for Brownian noise; (**c**) power spectral density (PSD) of the VLF band, with average slope (*β*); (**d)** PSD of ideal noise showing a slope of 0, −1 and −2 for white, pink and Brownian noise respectively; (**e**) detrended fluctuation analysis (DFA); (**f**) DFA of ideal noise showing a slope of 0.5, 1 and 1.5 for white, pink and Brownian noise respectively. Vertical bars represent standard error. Figures rendered with Matlab^41^.

To quantify the amount of structure and complexity in the beat interval signals, we employed multiscale entropy (MSE), a prominent entropy-based complexity measure^1^. This metric is based on sample entropy^16^, which quantifies the irregularity of a signal by estimating the negative logarithm of the probability that two similar sequences of length *m* in the signal are also similar for length *m* + 1. In MSE, sample entropy is calculated on multiple coarse-grained time series, which are constructed by averaging the data points within non-overlapping windows of increasing length, or “scale”. The sample entropy is then plotted as a function of scale, where higher scales represent longer-range temporal phenomena. Figure 2a,b show the MSE curve before and after ANS blockade, averaged over the entire dataset and compared to ideal noise processes. With both the ANS and SAN systems intact, it shows high values at all scales and minimal loss of complexity at the highest scales, as expected based on previous works^17,18^. Interestingly, in the autonomic blockade state, we can observe two phenomena: (a) significant loss of complexity in the low scales (up to about 10); (b) an invariant region in the high scales (above 10). To translate this result into a timescale of heart beats, recall that the MSE scales represent the number of averaged adjacent samples in the signal. In this case the samples are beat intervals, so an MSE scale of 11 corresponds to 12 heart beats (there are *N* + 1 beats for *N* intervals). The result therefore shows that, in canines, the SAN system regulates changes in the heart rate on time scales of roughly 12 heart beats or more.

To further probe the SAN and ANS effect on beat-interval signals, we additionally applied two fractal analysis methods (reviewed in^13^): detrended fluctuation analysis (DFA)^19^ and measurement of the fractal scaling exponent *β*^20^. Fractal objects are characterized by a self-similar structure in which each part has characteristics resembling the whole^21^. A *fractal temporal process* exhibits *statistical* self-similarity along its time and value axes^22–24^. The self-similar, or fractal, nature of HRV manifests in strong autocorrelations within the signal, that decay slowly with time (lag)^25^. The range of these autocorrelations is indicated by *β*, the slope of the log-log PSD in the VLF band^20^, and *α*, the slope of the log detrended fluctuation vs. log window size^19^. For beat-intervals, the DFA slope is usually calculated in two ranges corresponding to short- and long-term dynamics^26^. Following ANS blockade, we found a decrease in *β*, the PSD-based fractal coefficient, from an average of −0.7 ± 0.1 in the basal state to −1.2 ± 0.1 after ANS blockade (Fig. 2c,d). Short- and long-term DFA fractal scales, *α*_1_ and *α*_2_, were both 0.8 ± 0.1 and both increased to 1.3 ± 0.1 after ANS blockade (Fig. 2e,f). Moreover, not only has the DFA slope changed, but also the base level, i.e., the y-axis intercept of the curve (defined here for scale 4; see Methods). Due to the way DFA is calculated, the base-level corresponds mainly to the level of variance in the signal. After ANS blockade the base level was reduced substantially. The ANS contribution therefore increases the base level. This aligns both with the conclusion that the ANS contributes white noise to the beat intervals, and also with the observed increases in time-domain HRV metrics in the basal state.

The shape of the MSE curve and different values of *β* and DFA fractal scales are associated with various types of stochastic processes, such as white, pink, or Brownian noise, which can be characterized by their PSD (Fig. 2d). White noise is a random process characterized by a constant PSD at all frequencies. Pink (AKA flicker) noise is defined as having a PSD proportional to 1/*f*, while Brownian (AKA random-walk) noise is obtained by integrating white noise, and is characterized by a PSD proportional to 1/*f*^2^^27^. Thus, a slope of *β* = −1 corresponds to a 1/*f*process, i.e., pink noise, which also exhibits an MSE curve with high values at all scales (Fig. 2b,d). Similarly, a slope of *β* = −2 corresponds to a 1/*f*^2^, i.e. Brownian, noise process (Fig. 2d). The decreased slope in the SAN-only setting implies, again, that the beat intervals under this condition exhibit HRV metric values quantitively similar to Brownian noise values. The higher observed slope in the basal case implies that the ANS contribution increases the slope, and thus it shifts the beat interval dynamics towards white noise characteristics. DFA analysis further corroborates this. These shifts in *α*_1_ and *α*_2_ confirm the change in signal dynamics, from resembling pink noise in the basal state to resembling Brownian noise in the ANS blockade state (Fig. 2e,f).

### The ANS signature

**Figure 3:**
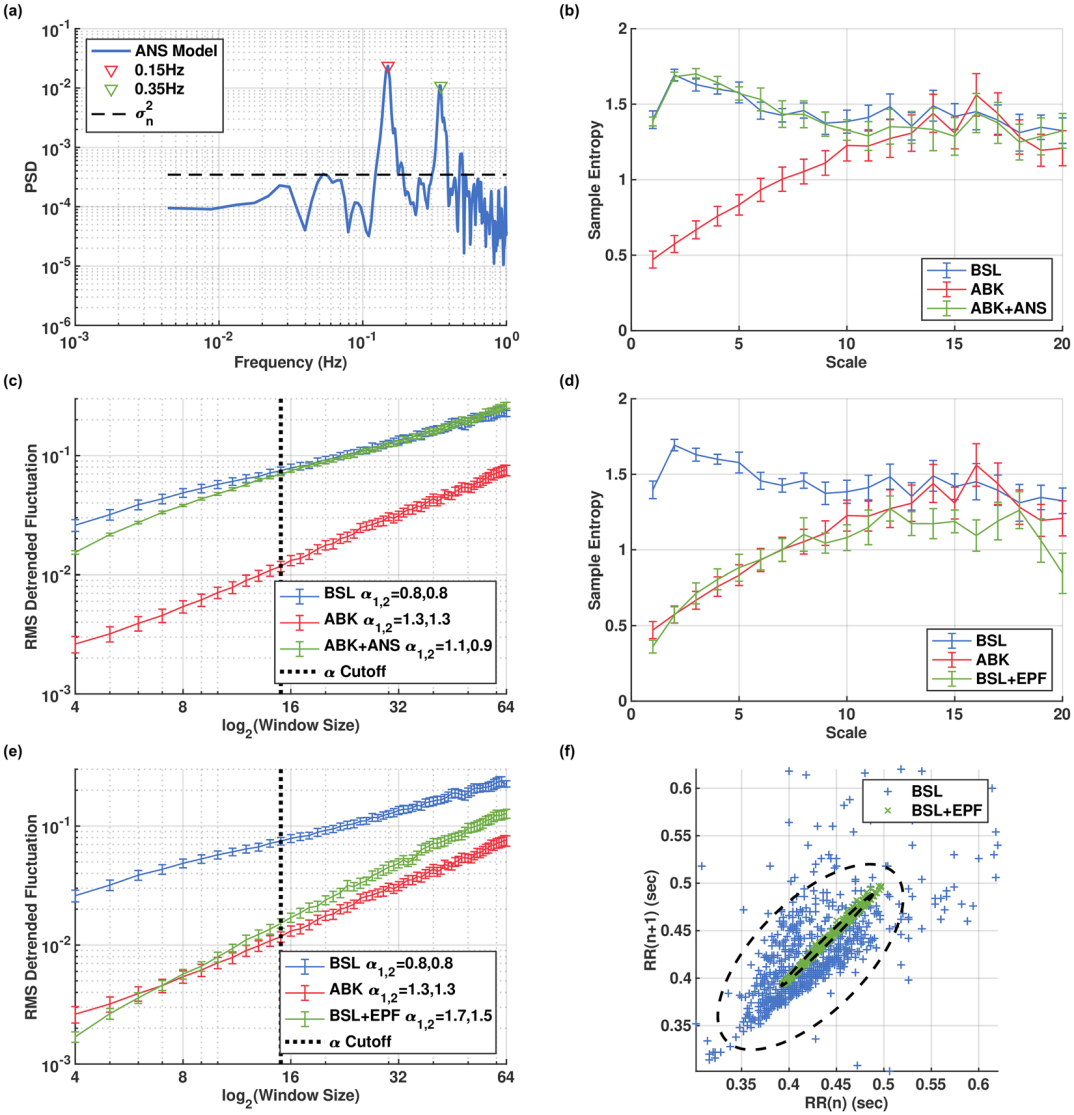
ANS and SAN signatures in canine basal ECG. (**a**) log power spectral density (PSD) of a generated signal from our model of the ANS contribution to beat intervals. Averaged basal (BSL), autonomic nervous blockade (ABK), and ABK with added samples from ANS model (ABK+ANS), showing dynamics shifted towards the BSL state: (**b**) multiscale entropy (MSE) and (**c**) detrended fluctuation analysis (DFA). BSL, ABK, and BSL after application of exponential filter (BSL + EPF), showing dynamics shifted towards the ABK state: (**d**) averaged multiscale entropy (MSE); (**e**) averaged detrended fluctuation analysis (DFA); (**f**) representative Poincaré plot. Vertical bars represent standard error. Figures rendered with Matlab^41^.

On the basis of our nonlinear HRV analysis results, we hypothesized that the ANS contribution to the beat interval time series might be modeled as a stochastic process with white noise characteristics. Such a signal could “add back” the missing low-scale MSE complexity and reduce the DFA slope, as observed in the denervated data. We also noted that the peaks in the LF and HF frequency bands of the PSD curve can be attributed to the ANS function because they were no longer present after ANS blockade, which is consistent with existing theory about their origins in regulation of respiration and blood pressure in humans^12,15^. Therefore, the ANS contribution should also have periodic components. To test these hypotheses, we created a model of the ANS contribution based on two periodic components, at frequencies corresponding to the respiratory and baroreceptor peaks, embedded in strong additive white noise with variance based on the basal state. Details of the model are provided in the Methods section. Note that this model aims to represent the ANS contribution to the beat-interval signal, not its actual physiological input to the SAN. The point is to identify which features of the beat-intervals are added by the presence of ANS regulation. Thus, while the SAN responds non-linearly (and therefore non-additively) to its actual physiological ANS input, here we employ a simplistic view which models the beat intervals as consisting of separate features contributed by each system. An example signal generated by the model is shown in Fig. 3a. Adding such a signal to an ANS blockade beat-interval signal produced a signal with MSE (Fig. 3b) curves closely resembling basal beat intervals (minimal and average MSE p-values were 0.23 and 0.71 across all scales, respectively), and DFA slopes shifted in the direction of the basal slopes (Fig. 3c). This indicates similar dynamics on all time scales, thereby demonstrating the model’s desired contribution. From these results (Fig. 3a–c) we conclude that the main features of ANS contribution are short-term time-domain HRV metrics, location of frequency peaks in the LF and HF bands, spectral power in these bands, low-scale MSE values, and DFA base-level.

### The SAN signature

The presented findings established that the SAN contributes mainly to the long-range patterns and very low frequencies within the beat intervals, while the ANS contributes mainly periodic regulation and short-term variance. On the basis of these findings, we can suggest various features which likely represent the contribution of the SAN to the beat-interval signal. Specifically, the most salient features are normalized power in the VLF band and in the “invariant” bands, high-scale MSE values and DFA slope. In an attempt to further isolate the SAN’s effect on the beat-interval signals, we searched for a strategy to obtain a beat-interval signal representing the denervated state from beat intervals in the basal state. Thus, we attenuated the ANS contribution by applying an exponentially decaying filter. The idea behind this filter is to enhance the lowest frequencies and apply a decaying attenuation to the rest of the spectrum (see Methods for details). The effect of this method can be observed in the MSE (Fig. 3d), DFA (Fig. 3e), and Poincaré (Fig. 3f) plots. The results show that the exponentially decaying filter causes a similar reduction in the low scales as is present in the ANS blockade state and is able to maintain most of the high-scale complexity (minimal and average MSE p-values were 0.05 and 0.5 across all scales, respectively). Therefore, in addition to the features defined above, SAN function could potentially be assessed based on basal ECG data by using such a filter or possibly more advanced signal processing techniques.

### Application to human data

**Figure 4:**
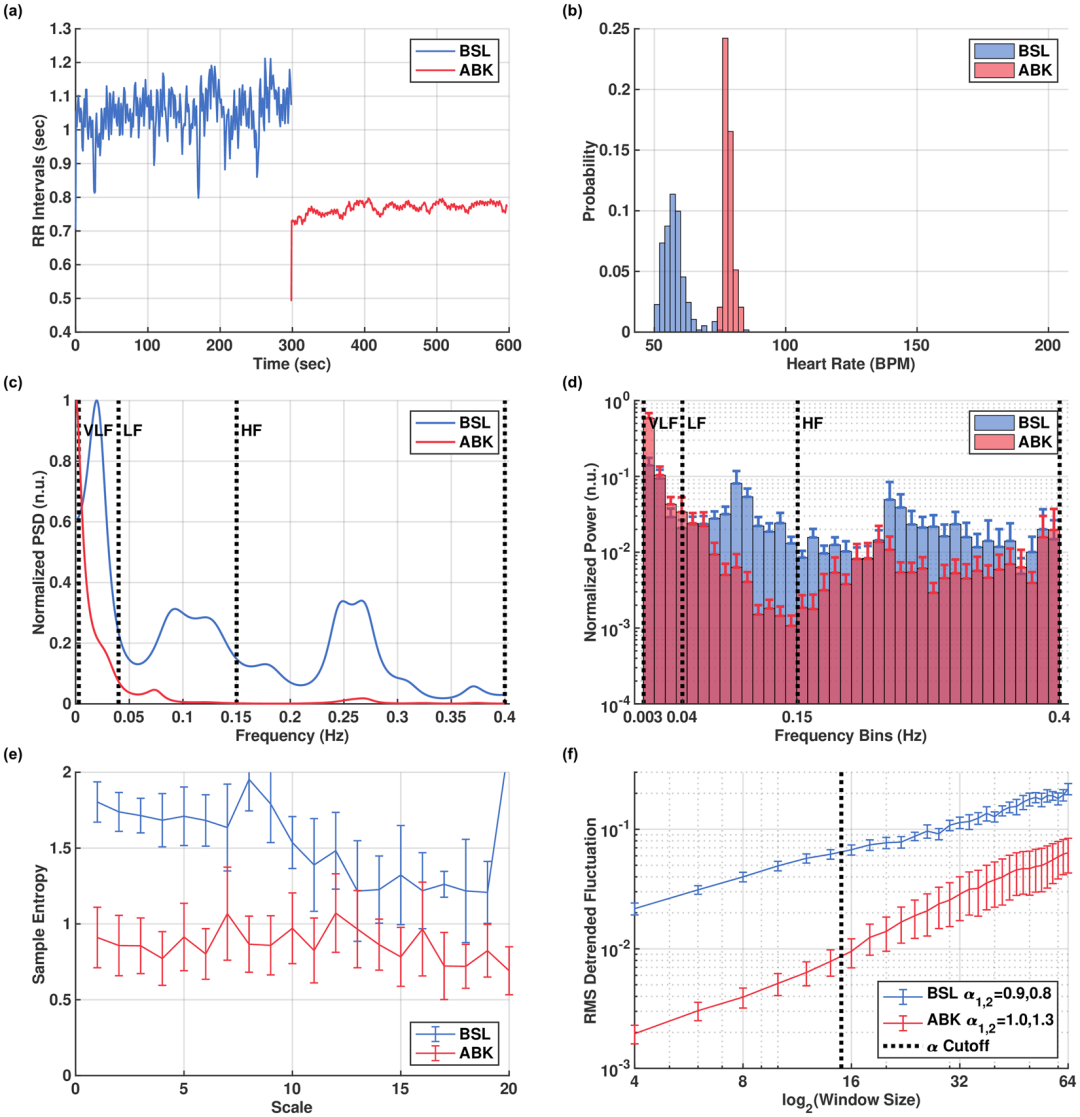
Human beat intervals in basal (BSL) and autonomic nervous blockade (ABK) states of the Ditor dataset. Representative examples of: (**a**) beat intervals; (**b**) beat interval distributions; (**c**) power spectral density (PSD), showing mostly-eliminated ANS-related peaks (baroreceptor, 0.1 Hz and respiratory, 0.25 Hz) after ABK. Averaged values of: (**d**) histogram of normalized PSD; (**e**) multiscale entropy (MSE); (**f**) detrended fluctuation analysis (DFA). Figures rendered with Matlab^41^.

**Figure 5:**
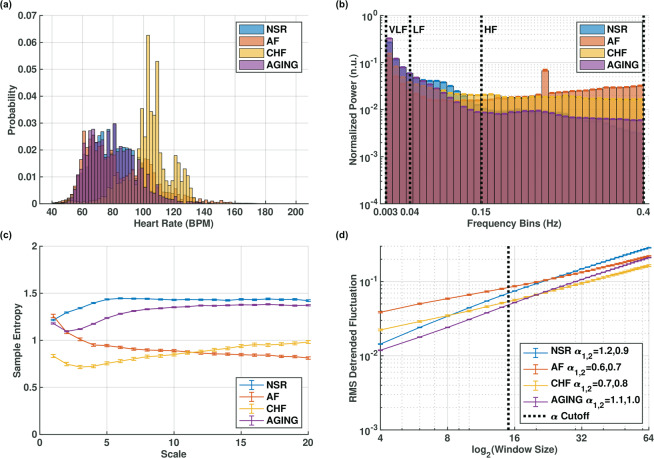
Human beat intervals of healthy (NSR), atrial fibrillation (AF), congestive heart failure (CHF), and aged individuals (AGING) from the PhysioNet datasets. (**a**) beat interval distributions; (**b**) histogram of normalized power; (**c**) multiscale entropy (MSE), showing the characteristic high-entropy at all scales in the NSR, a monotonic decrease indicating no SAN regulation in AF, a reduction with monotonic increase indicating impaired ANS and SAN regulation in CHF and slightly reduced complexity in AGING; (**d**) detrended fluctuation analysis (DFA), showing slopes in agreement with the MSE analysis. Vertical bars represent standard error. Figures rendered with Matlab^41^.

To evaluate the relevance of conclusions drawn from the canine data to humans, we obtained and analyzed two human datasets as follows: (a) A small dataset from Ditor *et al*.^28^, which includes basal (*ABL*; n = 3) and ANS blockade (*ABK*; n = 4) segments, each about 10 minutes long (Fig. 4). (b) A collection of large ECG databases from PhysioNet^29^ (Fig. 5), which include patients with atrial fibrillation (*AF*; n = 25), congestive heart failure (*CHF*; n = 8), and healthy subjects over the age of 60 (*AGING*; n = 47). We then compared these datasets to PhysioNet data from young healthy subjects (*NSR*; n = 25). The PhysioNet records from all groups were each at least 10 hours long. See methods for more information about these datasets.

HRV analysis results of the Ditor dataset are reported in Supplementary Table S1. A representative example of beat intervals before and after denervation is shown in Fig. 4a. As with the canines, ANS blockade increased the heart rate. Time-domain variability (Fig. 4b) decreased as expected, in accordance with the canine data. The characteristic respiratory peak in the high frequency (HF) band and the baroreceptor-reflex peak in the low frequency (LF) band are greatly attenuated after ANS blockade (Fig. 4c), but not completely abolished (compare to Fig. 1d of the canine case). This indicates that the denervation may have been partial, due to the doses or types of the administered drugs (see the AHA guidelines^30^) or other factors (see Limitations). However, as with the canines, the majority of spectral power is found in the VLF band after partial ANS blockade (Fig. 4d). The averaged MSE curves for this dataset (Fig. 4e) show substantial reduction in the lower scales after partial ANS blockade. However, there is also a reduction in the higher scales where we would expect an invariant region. The DFA curves (Fig. 4f) show a trend similar to the one observed in the canine data: the fractal slopes increased toward the Brownian noise value of 1.5 and the base level decreased.

In search for a way to further investigate the relevance of our conclusions regarding the relative contributions of the SAN and ANS to humans, we compared the MSE curves of young healthy humans to those of canines in the basal state, because MSE conveys both short- and long-range variability of beat interval dynamics. Figures 5c and 2a show that the healthy young humans and basal-state canines had comparable trends in their MSE curves. Specifically, the curves show high complexity at all scales, which is consistent with the trend expected for healthy humans^17^. Because it was previously shown that the human ANS contributes to the short-range variations^16^, and that both ANS and SAN contribute to HRV^9^, our current results, showing the similarity between human and canine MSEs, raise the possibility that similar mechanisms lead to short- and long-range variations in humans and canines alike. By also analyzing data from humans with AF and CHF we were able to explore this possibility.

The analysis results on the PhysioNet databases are summarized in Supplementary Table S2. We treat the healthy young subject group (*NSR*), containing subjects aged 20–45, as a control and compare the others to it. The first group we explored was the AF group. During AF, the heart beats are not generated by the SAN^31^. Thus, in AF, the SAN cannot regulate HRV, while the ANS might continue to affect it (albeit differently). Examining the results for the AF group, we found significantly increased time-domain variability in most metrics (Supplementary Table S2, Fig. 5a). Compared to the control, the AF group shows decreased VLF power combined with strong increases in both LF and HF power and no observable spectral peaks related to ANS regulation (Fig. 5b). The MSE curve for the AF group (Fig. 5c) shows a monotonically decreasing trend, a characteristic of white noise processes^1^ (Fig. 2b). The DFA curve (Fig. 5d) for the AF group further supports this finding, because it displays both a reduced slope, closer to the 0.5 value of white noise, and an increased base-level. Therefore, in presence of AF, the beat intervals do not exhibit their distinctive long-range patterns. The AF group has the highest entropy value on the lowest scale, and highest DFA base-level, both of which correspond to high-frequency noise content. These results, together with the fact that the SAN does not generate beats during an AF event, led us to conclude that long-range HRV regulation could be attributed to the SAN in humans, as for the canines.

Analysis of the CHF data shows a reduction in most time-domain beat interval variability measures (Supplementary Table S2; Fig. 5a). In the frequency domain, we observe decreased VLF power and increased HF power (Fig. 5b). MSE analysis shows reduced entropy at all scales (Fig. 5c). However, as opposed to AF, the trend is not monotonically decreasing, but mostly increasing in the high scales, similar to the Brownian noise case (Fig. 2b), which indicates long-range correlations in the beat intervals exist under CHF, but with reduced pattern complexity compared to NSR. The DFA slope is reduced but has a higher base-level (Fig. 5d) compared to NSR, indicating less fractal dynamics with more short-term variability. These findings, when combined with our conclusion from the AF case, and together with the established literature connecting ANS function to short-term HRV regulation^12,15^, can be interpreted as indicating reduced SAN activity and increased ANS activity under CHF.

Our third group included healthy subjects aged 60–76. Time-domain variability is reduced compared to the control group (Supplementary Table S2, Fig. 5a). Power in all bands decreased (Fig. 5b) but the general balance of power between the bands was maintained, with most power remaining in the VLF band. Both the MSE (Fig. 5c) and DFA curves (Fig. 5d) show that long-range correlations are maintained, albeit with a small reduction in complexity or self-similarity within the beat intervals. Based on our former conclusions regarding humans, these results imply a decline in SAN function due to aging.

### Estimating the degree of SAN clock-coupling by observing changes in HRV

**Figure 6:**
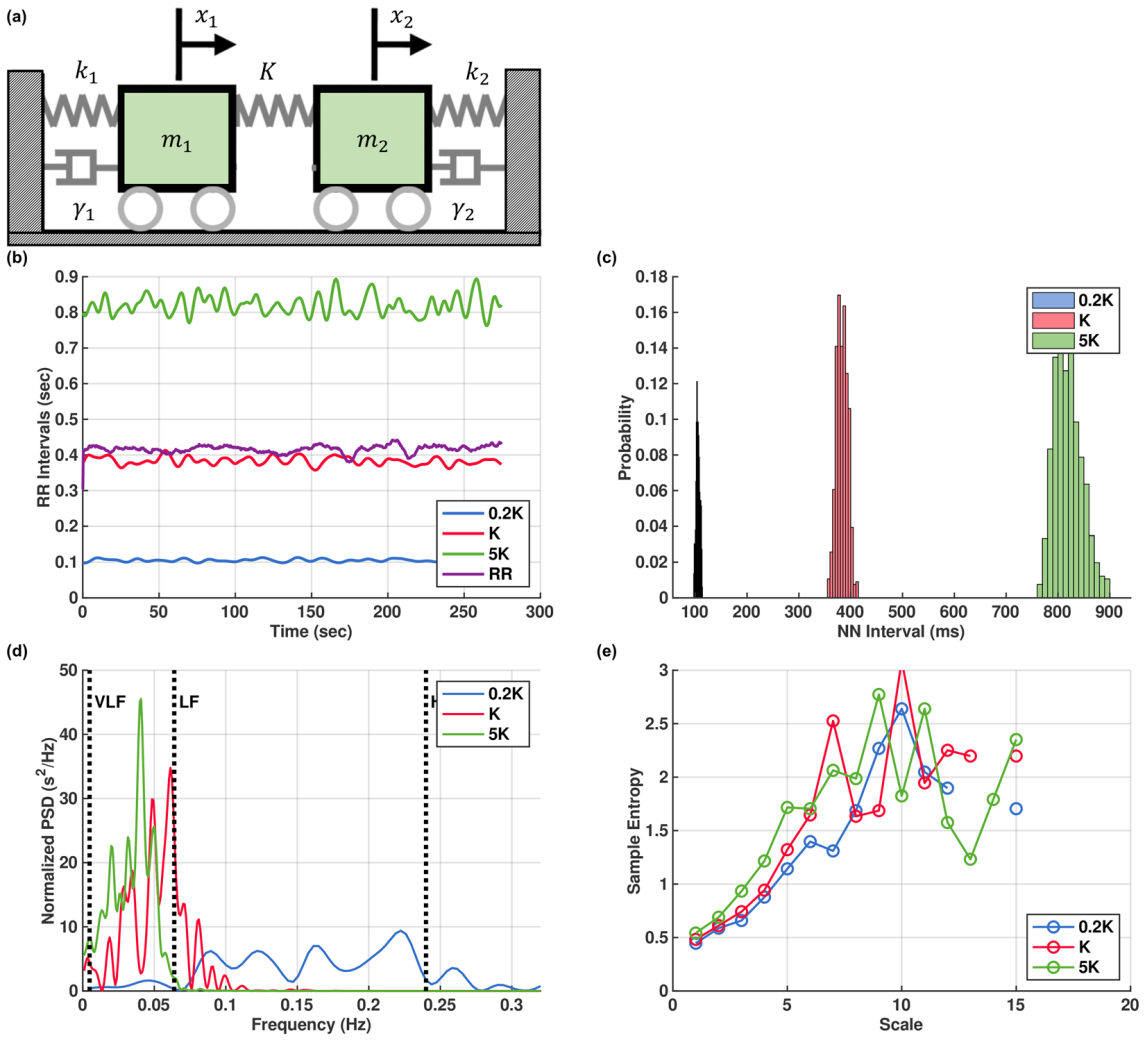
Simulation of a coupled-oscillator model fit to canine beat intervals, under different values of the coupling coefficient. (**a**) Schematic illustration of the model and its parameters; **(b**) beat intervals generated by the model with different coupling coefficient (***K***) values, compared to original canine intervals (RR); (**c**) beat interval distributions; (**d**) normalized power spectral density (PSD), showing increased VLF power with higher coupling; (**e**) multiscale entropy (MSE), showing generally higher values as coupling increases. The model parameters were fitted to canine beat intervals after autonomic blockade, and afterwards only the parameter ***K*** was changed by a constant factor to generate the plots. Figures rendered with Matlab^41^.

To provide mechanistic insight into how changes in the SAN’s coupled clock system can affect the SAN features present in the beat intervals, we used a mathematical model consisting of two mechanical oscillators (Fig. 6a; see further details in the methods). The two oscillators, each with a mass, dampening factor, spring and perturbing external force, represent the two SAN clocks: the first oscillator is the Ca^2+^ clock, which is regulated by local Ca^2+^ releases from internal Ca^2+^ storage. In previous experiments, reviewed in^7^, releases of Ca^2+^ are simulated as a spontaneous process with a rate identical to the heart rate. Thus, the intervals between Ca^2+^ releases are represented by movement of the first mass in our model. The second oscillator represents the ensemble of molecules on the cell membrane that can be affected by other external forces such as membrane current, or by spontaneous Ca^2+^ releases. The two oscillators are coupled by a spring with a constant *K*. Because the degree of coupling determines the SAN beating rate and the variability^32^, we changed the value of *K* to simulate changes in the degree of coupling and tested the effect on beat interval variability. Results show that the model can generate beat intervals comparable to the ANS blockade state in a representative canine sample (Fig. 6b). Moreover, as expected, an increase in *K* value increases the variability of the intervals. Reduction in the degree of coupling reduces the variability (Fig. 6c), VLF power (Fig. 6d) and entropy (Fig. 6e) compared to control (unmodified *K*). Conversely, increased coupling increases the variability and VLF power (Fig. 6c,e). This demonstrates that the degree of SAN clock-coupling could potentially be estimated by observing changes in frequency content and nonlinear HRV metrics.

## Discussion

Our first finding is that the SAN regulates the canine heart rate with complex long-range variations, while the ANS performs mainly short-term periodic regulation. These results were consistent in the time, frequency and non-linear analysis results presented in the previous section. Crucially, the non-linear methods show that the rich structure embedded in the beat intervals signal originates in the SAN, because high MSE values in high scales is a feature of signals with long-range correlations^17^. Such signals, e.g. 1/*f* (pink) noise contain the highest level of structure and complexity^33^ (Fig. 2b,d). When only the SAN system is intact, the MSE curve qualitatively resembles a Brownian noise curve (Fig. 2a,b): It increases monotonically and most of the complexity is found at the highest scale values, which correspond to the longest time-scales and the long-range variability in the beat intervals. Therefore, we conclude that most of the complexity in the canine beat interval signal, i.e., the richness of structure and information content, is a product of the SAN’s heart rate regulation.

Our frequency-domain results are in accordance with current theory, which maintains that the ANS system affects the heart rate at frequencies between 0.04-0.15 Hz and 0.15-0.4 Hz, known as the LF and HF bands^4,12,15^. However, these prevailing theories regarding frequency-domain HRV fail to explain the origin of power in lower frequencies, such as in the VLF band (0.003-0.04 Hz)^12^. These lower frequencies, corresponding to long-term regulation, account for the vast majority of spectral power within the beat-interval signal. While some recent works show evidence that the VLF power is in fact an intrinsic property of the heart itself^34^, this view is not widely held and still controversial. Our results strengthen this theory.

Our second finding is that the ANS contribution to the beat-interval signal might be modeled as a stochastic process with white noise characteristics and periodic components at frequencies corresponding to the respiratory and baroreceptor peaks. MSE analysis of basal and ANS canine data revealed an important finding regarding the ANS’s contribution to the beat interval signal. The reduction in the low scales present under denervation indicates that the ANS affects mainly these scales, which correspond to short-term variability. It has been demonstrated, both theoretically and empirically, that white noise has a monotonically decreasing MSE curve^17^ (Fig. 2b). This suggests that the ANS may contribute considerable uncorrelated noise, e.g. white noise, to the beat intervals. Such a contribution “averages out” under the coarse-graining part of the MSE operator as scale increases; thus, its effect decreases with scale. Together with the monotonically increasing, Brownian-like curve present in the denervated data, a flat, 1/*f*-like, curve is created as in the basal case. The DFA analysis we performed further corroborated this analysis because the slopes shift towards white noise (*α* = 0.5) in the presence of the ANS. To our knowledge no previous model of the ANS contribution to the heart rate intervals was suggested. However, in a previous study, Kuusela *et al*. modeled the beat-interval time series as a one-dimensional stochastic difference equation^35^. Interestingly, their model consisted of combining a deterministic nonlinear component and a stochastic component modeled as Gaussian white noise. They concluded that the model accurately predicts the complex dynamics of the human heart rate and, moreover, that the stochastic white noise component is an integral part of these dynamics—corroborating our model of ANS contribution.

Our third finding is a set of features for both the ANS and the SAN which can be identified in beat intervals obtained from regular canine ECG, i.e. without need for ANS blockade. The SAN-related features can be identified by calculating the (a) normalized power in the VLF band; (b) normalized power in the “invariant” bands; (c) high-scale MSE values and (d) DFA slope. The ANS-related features we identified are (a) short-term time-domain HRV metrics (SDNN, RNSSD, pNN, SD1), (b) location of frequency peaks in the LF and HF bands, (c) spectral power in these bands, (d) low-scale MSE values and (e) DFA base-level. Note that in all cases the locations of the frequency bands and the exact MSE scales considered as low or high are mammal specific. Behar *et al*. have previously suggested a method of determining the frequency bands for different mammals, which can also be applied in this case^14^.

Our fourth finding is evidence supporting the hypothesis that our conclusions based on the canine data are also applicable to humans. Despite the drawbacks of the human double blockade dataset we used (Ditor *et al*.^28^), i.e., its limited size and the fact that it may not represent a full ANS blockade, none of the results obtained from it contradict any of the conclusions drawn from the analysis of the canine data. These data suggest that the SAN system contributes mainly to the long-range, low-frequency changes in heart rate variability, even in humans. Moreover, the results from the much larger second human dataset, taken from PhysioNet, further supported our hypothesis with regards to the relative contributions of the SAN and ANS.

Our fifth finding is insight into the function of the SAN and ANS during pathological and aging processes in humans. Our HRV analysis of the PhysioNet datasets, when taken together with previous works dealing with AF, CHF and aging, provides both further evidence for the relative contributions of the ANS and SAN, as well as an understanding of these conditions in terms of these two discrete heart rate regulatory systems. The AF data did not exhibit the characteristic SAN features that we defined: the beat-intervals lacked long-range patterns and were instead characterized by increased short-term variance and high-frequency power. This is consistent with our theory because, as explained, during AF, beat intervals are not generated exclusively by the SAN but by abnormal electrical activity originating in the atria, which causes erratic beat-intervals^31^. Our MSE results are highly consistent with Costa *et al*.^18^ which also analyzed human AF patients; however, our analysis further provides mechanistic insight, specifically that SAN regulation is impaired in this case. The DFA curve for the AF group further supports this observation, because it displays both a reduced slope, closer to the 0.5 value of white noise, and an increased base-level.

CHF is associated with failure of SAN function in conjunction with ANS compensation^36^. Our findings on the CHF data support this observation. We show decreased VLF power, increased HF power, reduced entropy at all scales, and a reduced DFA slope, but with a higher base-level. These results are all consistent with various other works studying different HRV metrics under CHF^18,37,38^, and can be interpreted as indicating reduced SAN activity and increased ANS regulation during CHF.

A decrease in complexity of beat intervals was previously associated with aging^18^. Indeed, both our MSE and DFA curves are consistent with this, as they show that long-range correlations are maintained, albeit with a reduction in complexity or self-similarity within the beat intervals. We have also shown a reduction in spectral power, consistent with, e.g., Kuo *et al*.^39^. Our results are also in agreement with Yaniv *et al*.^10^, who observed loss of pacemaker function in aged mice. Although these authors obtained similar results, we are able to provide further insight: when considering the SAN and ANS features discussed previously, we conclude that both the ANS (low-scale MSE) and SAN function (high-scale MSE) are reduced by aging. General loss of complexity, and therefore a reduction in HRV, is present as a result of both systems together. The decrease of power in all bands with age shows, again, that both the ANS, with its periodic components in the LF and HF bands, and the SAN, the main contributor to VLF power, are functionally reduced as age increases.

### Limitations

Our discussion focused exclusively on the relative contributions of the ANS and SAN, which have been demonstrated to be the two main systems controlling the beat intervals. However, additional factors, such as hormones, may also modulate the beat interval’s short- and long-range patterns *in vivo*^40^. Such factors are likely present after ANS blockade, for example, due to residual blood hormone levels. However, interval dynamics similar to the denervated case were also shown in isolated mice and rabbit SAN tissue^2,10^, and in the case of isolated SAN tissue, hormonal factors do not exist. Therefore, in our analysis, we assume that these factors have minimal effects as compared to those of the ANS and SAN systems.

## Methods

We implemented all data processing and analysis tasks related to this paper as an open-source software platform, *PhysioZoo*^13^, which incorporates *mhrv*, an algorithmic heart rate variability (HRV) toolbox for Matlab^41^ It can be easily adapted to work with both human and animal data. Specifically, PhysioZoo was used for ECG peak detection and segmentation, generating RR-intervals, ectopic beat removal, calculating HRV metrics and applying various filters to RR interval signals. We refer the reader to the supplemental material of the PhysioZoo paper^13^. which contains comprehensive descriptions of the algorithms and their implementation details. The PhysioZoo platform is freely available at https://physiozoo.com.

### Canine data

Electrocardiographic data from n = 27 canines was obtained from Billman *et al*.^11^. The protocols and experimental procedures were approved by the original research committee; for further experimental details, see the aforementioned paper. Each recording included a basal segment, followed by administration of atropine (50 μg/kg, i.v.) and finally administration of propranolol (1.0 mg/kg, i.v). In some cases, the drugs were administered in reverse order. Each segment we considered was at least 5 minutes in duration. We extracted two ECG segments from each record: a basal segment, before any drugs were administered, and a double-blockade segment, after both atropine and propranolol were administered. By extracting the first and third segment from each record (i.e., before and after administration of both drugs), we obtained a basal and double-blockade segment from each record. Visual inspection of the data revealed that following each drug being administered, there was a short (up to 20-second) transient period until the heart rate reached a steady state again. Thus, transient periods were removed from all segments before analysis, as follows: the value of a 10-second sliding window lowpass filter was compared to a threshold taken as the mean value of the segment starting from 30 seconds after the start of the segment (this part of the segment was considered transient-free). When the sliding window average was within 5% of the threshold, the transient period was considered over, and the rest of the segment was used. After transient removal, each segment was trimmed at the end to standardize the duration of the segments to 5 minutes.

### Human data

Electrocardiographic data were obtained from Ditor *et al*.^28^ (n = 3) and from the following publicly-available PhysioNet^29^ databases: *nsrdb*, *nsr2db*, *afdb* and *chfdb*. A total of 25 records from subjects aged 20–45 were used from *nsrdb* and *nsr2db*. We collectively referred to this as our NSR dataset. All 25 records from *afdb* were used (our AF dataset). A total of 8 records of subjects aged 22–45 were used from *chfdb* (our CHF dataset). Furthermore, we created the AGING dataset from a total of 47 records of healthy, arrhythmia-free subjects aged 60–76 from *nsrdb* and *nsr2db*. Each record was split into analysis windows of 10-minute duration (PhysioNet databases) or 5-minute duration (Ditor dataset). This was chosen as a tradeoff to ensure a duration long enough to obtain data points for long-range correlation analysis (multi-scale entropy and detrended fluctuation analysis) but also short enough for frequency-domain analysis, which assumes stationarity of the data.

### RR interval time series construction and filtering

An RR interval is the time difference between two adjacent R-peaks in an ECG signal. Given a series of *N* R-peak detection times ${t}_{0},{t}_{1},\ldots ,{t}_{N-1}$, the corresponding RR interval time series is defined as

$${\rm{RR}}({t}_{i})={t}_{i+1}-{t}_{i},i=0,1,\mathrm{..}.,N-2.$$

We used the PhysioZoo platform^13^ for R-peak detection and construction of the RR interval time series. The platform implements ECG peak detection for human data and allows adaptation of the detector parameters for other mammalian data.

After construction, the RR intervals are filtered such that out of place, or *ectopic*, beats are removed. The aim is to keep only intervals for which the probability is high that both of their beats originate in the sinoatrial node (SAN), which are known as NN (normal-normal) intervals^42^. PhysioZoo’s *mhrv* toolbox implements three types of RR interval filtering techniques, based mostly on previous works^42–46^: (a) range-based filtering (RBF), in which intervals of duration longer or shorter than threshold values are discarded; (b) moving-average filtering (MAF), in which an interval is removed if its value is greater or smaller than the average of its neighboring intervals by some percentage; and (c) quotient filtering (QF), in which an interval is removed if it varies by more than some percentage from its previous or successive interval. Consult the PhysioZoo supplemental material^13^ for further details.

### Human RR interval processing

The PhysioNet datasets we analyzed contain long-term ambulatory ECG recordings of at least 10 hours each. Thus, records typically include segments of noise or artifacts due to movement of the subject or measurement device (Holter monitor). Such segments are best removed for the sake of accurate heart rate variability (HRV) analysis^12^. Therefore, after the HRV metrics in each analysis window were calculated, the mean and standard deviations of the RR intervals, *μ*_*RR*_ and *σ*_*RR*_ respectively, were compared to threshold values *RR*_min_ and *RR*_max_ to ensure that the window contains mostly valid data and not, e.g., noise or artifacts. An analysis window was discarded if:

$$\begin{array}{c}{\mu }_{RR}+2{\sigma }_{RR} > R{R}_{{\rm{\max }}},\,{\rm{or}}\,\\ {\mu }_{RR}-2{\sigma }_{RR} < R{R}_{{\rm{\max }}}\end{array}$$

In other words, assuming a normal distribution of RR intervals, an analysis window was discarded if roughly more than 5% of its intervals were above or below the respective threshold. In practice, we used threshold values of *RR*_max_ = 1.2 and *RR*_min_ = 0.5 seconds, which correspond to heart rates of 50 BPM and 120 BPM respectively. We chose conservative values to prevent analysis of segments which are either noisy or contain a lot of or movement. This filtering method removed 5.9%, 14.6%, 12.1% and 7.2% of the analysis windows from the NSR, AF, CHF and AGING datasets respectively. Within the windows themselves, the RR intervals were filtered out with the RBF, with values of *RR*_min_ and *RR*_max_ as above.

For the Ditor dataset we performed no window removals due to the scarcity of the data. The intervals in each window were filtered with RBF using *RR*_max_ = 1.5 and *RR*_min_ = 0.5.

### Canine RR interval processing

When analyzing the canine data, we used all three interval filtering methods described above, with the following configuration: (a) RBF: We rejected intervals outside the range of 0.3–1.2 seconds, corresponding to heart rates outside the range 50–200 BPM. (b) MAF: We used a window size of 21 samples (10 samples on each side of the central sample) and filtered out the sample if its value exceeded 40% of the window’s average. (c) QF: We used a value of *r* = 0.8, corresponding to an 80% tolerance when deciding to filter an interval based on its predecessor or successor.

### HRV analysis

Filtered intervals were analyzed with PhysioZoo to obtain HRV metrics per analysis window. All supported metrics were calculated, including time domain, frequency domain and nonlinear methods. Parameters of HRV metrics were adapted to canines where relevant, based on our previous works^13,14^.

### Detrended Fluctuation Analysis (DFA) slope

DFA^26^ is a method of assessing the dynamics of a signal. The slope of a DFA plot indicates the pattern of long-range correlations within it. White noise, the most random type of process, exhibits no long-range correlations and has a slope of *α*_w_ = 0.5. The slope of a pink noise process, which contains complex patterns and correlations on multiple time scales^33^, has a slope of *α*_p_ = 1.0. Many physiological processes, particularly RR intervals, are known to display these type of dynamics^20^. The slope of a Brownian noise process, which we have shown to have dynamics similar to RR intervals under ANS blockade, is *α*_b_ = 1.5 (Fig. 2f). We tested two features of the DFA plot: its slope and its base level. The slope was calculated over two scale ranges, 4–15 and 16–64, corresponding to short and long-term dynamics^19^. We defined the DFA base level as the value of the shortest scale (n = 4) we used. Note that by definition of the DFA, its value is zero at scales 1 and 2, i.e., *F*(1) = *F*(2) = 0. Thus, only *n* = 3 could be a smaller possibility for the base level definition.

### Model of ANS contribution to the RR intervals

The ANS contribution model consists of generating a process of two sine waves embedded in white noise. This process is then sampled at the times of an ANS blockade beat-interval signal, and the result is then mixed in with that signal. We compare the resulting signal’s dynamics with the basal setting. Given an RR interval time series $RR(t),\,t\,{\epsilon }\,[0,\,T]$, the ANS model is generated as follows:

1. Create a signal *x*(*t*) from two sine waves with frequencies *f*_1_ and *f*_2_:
  $$x(t)=\,\sin (2\pi {f}_{1}t)+\,\sin (2\pi {f}_{2}t),t\in [0,T].$$
2. Define the desired variance of *x*(*t*) according to the RR intervals:
  $${\sigma }_{x}^{{\prime} 2}={\sigma }_{r}^{2}{\alpha }_{m}^{2}$$
  where ${\sigma }_{r}^{2}$ is the variance of *RR*(*t*) and *α*_*m*_ is the “mix ratio”, a parameter which specifies the contribution of the model relative to the RR intervals.
3. Scale *x*(*t*) to the desired variance:
  $${x}_{s}(t)=(x(t)-{\mu }_{x})\frac{{\sigma }_{x}^{{\prime} }}{{\sigma }_{x}}$$
  where *μ*_*x*_ is the mean and *σ*_*x*_ is the standard derivation of *x*(*t*) before scaling.
4. Define the desired variance of the noise, relative to the variance of the sine waves:
  $${\sigma }_{n}=\frac{1}{SN{R}_{n}}{\sigma }_{x}^{{\prime} }$$
  where *SNR*_*n*_ is a parameter which specifies the signal-to-noise ratio between the additive noise and the *x*(*t*).
5. Generate a Gaussian white noise process *n*(*t*) with zero mean and variance ${\sigma }_{n}^{2}$.

The resulting model of ANS contribution is

$$y(t)={x}_{s}(t)+n(t),\,t\in [0,T].$$

Based on frequency-domain HRV analysis, we selected *f*_1_ = 0.15 and *f*_2_ = 0.35 Hz as the sine frequencies, corresponding to the average peak locations in the LF and HF bands of the canine data. We empirically selected the mix ratio *α*_*m*_ = 0.5 and the signal to noise ratio $SN{R}_{n}=2$.

### Exponentially decaying filter

The goal of this filter is to strengthen the hypothesis that the SAN contributes mainly to the long-range patterns and low frequencies within the RR intervals. To that end we seek to apply a transformation to basal data in order to remove some or most of the ANS contribution, such that the resulting signal resembles (in terms of its dynamics) denervated data.

The filter is a simple one-sided decaying exponent in the time domain, i.e., $h(t)=u(t){e}^{-|\alpha |t}$ where *u*(*t*) is a unit step at zero. The *α* parameter can be thought of as a decay-rate constant. The frequency-domain equivalent of such a filter is $H(f)=1/(|\alpha |+j2\pi f)$. Thus, it enhances the lowest frequencies and applies a decaying attenuation to the rest of the spectrum. The decay-rate constant controls how much of a “memory” the filter has. The smaller the decay rate, the more of the signal the filter will see at each sample. The downside of this filter is that it has a nonlinear phase, meaning it creates uneven distortions of the signal. We empirically set the decay-time constant such that the exponent decays to a value of 10^−3^ within 75 seconds.

### Computational modeling

The coupled oscillator model consists of two masses, two damping elements, and three springs (two system springs and one coupling spring). Additionally, an external force is applied to the first mass. The first oscillator represents the Ca^2+^ clock^7^ under the following assumptions:

1. The external force works directly on the first mass.
2. The external force represents spontaneous Ca^2+^ releases, modeled as a pulse train with a frequency roughly corresponding to dog heart rate.
3. Ca^2+^ releases can occur spontaneously without any feedback from the other coupled oscillator model components.
4. The first mass displacement represents the interval between global spontaneous Ca^2+^ releases.
5. The rate of global spontaneous Ca^2+^ releases is similar to the rate of spontaneous firing rate of pacemaker cells^47^.

The second oscillator represents the membrane clock, an ensemble of channels, pumps and exchangers, under the following assumptions:

1. Two components affect the membrane clock, external force (ANS) and the Ca^2+^ clock through the coupling factor (*K*).
2. The clock rate is similar for Ca^2+^ and membrane clocks.
3. The second mass displacement represents the self-oscillated pacemaker interval.

A coupled oscillator model can be modeled by the following system of differential equations:

$$\begin{array}{rcl}{m}_{1}{\ddot{x}}_{1} & = & -{\gamma }_{1}{\dot{x}}_{1}-{k}_{1}{x}_{1}-K({x}_{1}-{x}_{2})+{F}_{1}(t)\\ {m}_{2}{\ddot{x}}_{2} & = & -{\gamma }_{2}{\dot{x}}_{2}-{k}_{2}{x}_{2}-K({x}_{2}-{x}_{1})+{F}_{2}(t)\end{array}$$

where *m*_*i*_ represents the mass of each oscillator, *γ*_*i*_ is the dampening factor, *k*_*i*_is the spring coefficient, *F*_*i*_(*t*) is an external force applied to each system and *K* is the coupling factor.

Due to the above assumptions, we’re interested in modeling the movement of the second mass as a function of the force on the first mass. Taking a Laplace transform of the first equation above:

$${X}_{1}(s)=\frac{K{X}_{2}(s)+{F}_{1}(s)}{{m}_{1}{s}^{2}+{\gamma }_{1}s+{k}_{1}+K}$$

Similarly, for the second equation, assuming *F*_2_(*t*) = 0,

$${X}_{2}(s)({m}_{2}{s}^{2}+{\gamma }_{2}s+{k}_{2}+K)=K{X}_{1}(s).$$

By plugging *X*_1_(*s*) into the above equation and rearranging, we obtain the following transfer function:

$$H(s)=\frac{{X}_{2}(s)}{{F}_{1}(s)}=\frac{K}{({m}_{2}{s}^{2}+{\gamma }_{2}s+{k}_{2}+K)({m}_{1}{s}^{2}+{\gamma }_{1}s+{k}_{1}+K)-{K}^{2}}$$

We used the Matlab^41^ System Identification Toolbox to estimate the parameters of the above transfer function based on one of the canine ANS blockade records. The output of the system was set to be the beat interval signal calculated from the record. We provided an input pulse train at a frequency corresponding to the heart rate estimated from the canine data. A noisy pulse train with varying pulse height was created by empirically combining Gaussian white noise both additively ($\mu =0,{\sigma }^{2}=0.0025$) and multiplicatively $(\mu =1,{\sigma }^{2}=0.04)$.

Using the estimated transfer-function parameters, we simulated the system with an input force *F*_1_(*t*) equal to a pulse train of the same frequency (but without added noise), initial conditions equal to zero, and ${F}_{2}(t)=0$. The system was simulated by converting it to a system of first-order differential equations:

$$\begin{array}{rcl}{v}_{1} & = & {\dot{x}}_{1}\\ {v}_{2} & = & {\dot{x}}_{2}\\ {m}_{1}{\dot{v}}_{1} & = & -{\gamma }_{1}{v}_{1}-{k}_{1}{x}_{1}-K({x}_{1}-{x}_{2})+{F}_{1}(t)\\ {m}_{2}{\dot{v}}_{2} & = & -{\gamma }_{2}{v}_{2}-{k}_{2}{x}_{2}-K({x}_{2}-{x}_{1})\end{array}$$

and solving numerically for each time step with a sampling frequency of *f*_*s*_ = 25 *Hz*. To test the effect of *K*, the calculated coupling coefficient, we repeated the simulation with values of 0.2*K* and 5*K*.

## Data and materials availability

All code implementing the HRV analysis algorithms is freely available at https://physiozoo.com. The data analysis code, and a link to the datasets will be made freely available on github following publication of the paper.

## Supplementary information

Supplementary Materials.
