# Supplementary material for: Signatures of the autonomic nervous system and the heart’s pacemaker cells in canine electrocardiograms and their applications to humans
Source: Sci Rep. 2020 Jun 19;10:9971. doi: 10.1038/s41598-020-66709-z (PMC7305326; doi:10.1038/s41598-020-66709-z)
Supplement: Supplementary file 1 — Supplementary Materials. [file 41598_2020_66709_MOESM1_ESM.docx]

Supplementary Materials for

Signatures of the autonomic nervous system and the heart’s pacemaker cells in canine electrocardiograms and their applications to humans

Aviv A. Rosenberg, Ido Weiser-Bitoun, George E. Billman and Yael Yaniv^*^

*Correspondence to: [yaely@bm.technion.ac.il](mailto:yaely@bm.technion.ac.il)

|  | | **BSL** | **ABK** | |
| --- | --- | --- | --- | --- |
| **Time Domain** | |  |  | **p-value** |
| AVNN | (ms) | 1145.7 ± 35.7 | 714.2 ± 33.2 * | 0.00 |
| SDNN | (ms) | 62.4 ± 4.2 | 21.2 ± 9.5 * | 0.00 |
| RMSSD | (ms) | 67.4 ± 11.7 | 5.5 ± 1.1 * | 0.00 |
| pNN50 | (%) | 43.8 ± 11.2 | 0.0 ± 0.0 | 0.01 |
| SD1 | (ms) | 47.8 ± 8.3 | 3.9 ± 0.8 * | 0.00 |
| SD2 | (ms) | 70.7 ± 5.2 | 29.2 ± 13.2 * | 0.00 |
| **Frequency Domain** | |  |  |  |
| HF Power | (ms^2^) | 718.9 ± 180.0 | 25.8 ± 16.5 | 0.01 |
| HF Norm. | (n.u.) | 38.7 ± 9.5 | 12.0 ± 3.9 | 0.13 |
| HF Peak | (Hz) | 0.2 ± 0.0 | 0.3 ± 0.0 | 0.05 |
| LF Power | (ms^2^) | 509.7 ± 110.3 | 53.3 ± 45.6 * | 0.01 |
| LF Norm. | (n.u.) | 29.7 ± 6.0 | 9.7 ± 2.9 | 0.05 |
| LF Peak | (Hz) | 0.1 ± 0.0 | 0.1 ± 0.0 | 0.98 |
| VLF Power | (ms^2^) | 467.4 ± 83.7 | 433.4 ± 398.2 * | 0.00 |
| VLF Norm. | (n.u.) | 28.0 ± 5.5 | 53.9 ± 2.7 * | 0.01 |
| LF/HF | (n.u.) | 1.4 ± 0.5 | 1.7 ± 0.4 | 1.00 |
| Tot. Power | (ms^2^) | 1748.6 ± 227.3 | 706.3 ± 640.0 * | 0.00 |
| **Nonlinear** | |  |  |  |
| $\beta$ | (n.u.) | -0.9 ± 0.2 | -1.6 ± 0.2 | 0.08 |
| $\alpha_{1}$ | (n.u.) | 0.9 ± 0.1 | 1.0 ± 0.1 | 0.88 |
| $\alpha_{2}$ | (n.u.) | 0.8 ± 0.1 | 1.3 ± 0.1 | 0.05 |
| SampEn | (n.u.) | 1.8 ± 0.1 | 0.9 ± 0.2 | 0.04 |
| MSE5 | (n.u.) | 1.7 ± 0.2 | 0.9 ± 0.2 | 0.19 |
| MSE10 | (n.u.) | 1.5 ± 0.2 | 1.0 ± 0.2 | 0.53 |
| MSE15 | (n.u.) | 1.3 ± 0.3 | 0.8 ± 0.2 | 0.19 |
| MSE20 | (n.u.) | 2.2 ± 0.0 | 0.7 ± 0.1 | 0.75 |

Table S1: Heart rate variability (HRV) analysis results for the Ditor human dataset under the basal (BSL) and autonomic blockade (ABK) states. See main text for definition of each metric and analysis parameter. Reported frequency domain metrics are based on autoregressive (AR) modeling. Results reported as mean ± standard error (SE). A total of $N=6$ segments were analyzed from all records combined; an asterisk denotes $p<0.01$ in paired t-test.

|  | | **NSR (**$\boldsymbol{N=3156}$**)** | **AF (**$\boldsymbol{N=1173}$**)** | **CHF (**$\boldsymbol{N=946}$**)** | **AGING (**$\boldsymbol{N=5809}$**)** |
| --- | --- | --- | --- | --- | --- |
| **Time Domain** | |  |  |  |  |
| AVNN | (ms) | 791.6 ± 2.3 | 786.6 ± 4.3 | 606.4 ± 3.0 * | 797.0 ± 1.7 |
| SDNN | (ms) | 69.0 ± 0.5 | 92.7 ± 1.7 * | 58.1 ± 1.3 * | 55.7 ± 0.4 * |
| RMSSD | (ms) | 37.9 ± 0.4 | 122.8 ± 2.5 * | 65.6 ± 1.8 * | 34.1 ± 0.4 * |
| pNN50 | (%) | 13.0 ± 0.2 | 33.6 ± 0.9 * | 4.2 ± 0.2 * | 5.5 ± 0.1 * |
| SD1 | (ms) | 26.8 ± 0.3 | 86.9 ± 1.8 * | 46.4 ± 1.3 * | 24.1 ± 0.3 * |
| SD2 | (ms) | 93.1 ± 0.7 | 96.0 ± 1.7 | 65.4 ± 1.5 * | 73.6 ± 0.5 * |
| **Frequency Domain** | |  |  |  |  |
| HF Power | (ms^2^) | 393.2 ± 13.6 | 3114.6 ± 114.1 * | 1410.5 ± 81.6 * | 343.2 ± 13.1 * |
| HF Norm. | (n.u.) | 16.3 ± 0.2 | 59.4 ± 0.6 * | 42.0 ± 0.7 * | 17.1 ± 0.2 * |
| HF Peak | (Hz) | 0.3 ± 0.0 | 0.3 ± 0.0 * | 0.3 ± 0.0 * | 0.3 ± 0.0 * |
| LF Power | (ms^2^) | 663.3 ± 10.6 | 1031.3 ± 39.8 * | 755.5 ± 39.2 * | 393.2 ± 8.1 * |
| LF Norm. | (n.u.) | 32.2 ± 0.2 | 17.4 ± 0.3 * | 26.0 ± 0.3 * | 25.0 ± 0.2 * |
| LF Peak | (Hz) | 0.1 ± 0.0 | 0.1 ± 0.0 * | 0.1 ± 0.0 * | 0.1 ± 0.0 * |
| VLF Power | (ms^2^) | 1054.0 ± 20.9 | 657.1 ± 31.1 * | 577.8 ± 36.9 * | 692.7 ± 11.3 * |
| VLF Norm. | (n.u.) | 42.8 ± 0.3 | 19.2 ± 0.5 * | 27.2 ± 0.6 * | 46.5 ± 0.2 * |
| LF/HF | (n.u.) | 3.3 ± 0.0 | 0.4 ± 0.0 * | 1.1 ± 0.0 * | 2.9 ± 0.0 * |
| Tot. Power | (ms^2^) | 2357.8 ± 40.8 | 4930.1 ± 167.3 * | 2821.1 ± 139.6 * | 1639.4 ± 28.3 * |
| **Nonlinear** | |  |  |  |  |
| $\beta$ | (n.u.) | -0.7 ± 0.0 | -0.6 ± 0.0 * | -0.5 ± 0.0 * | -0.8 ± 0.0 * |
| $\alpha_{1}$ | (n.u.) | 1.2 ± 0.0 | 0.6 ± 0.0 * | 0.7 ± 0.0 * | 1.1 ± 0.0 * |
| $\alpha_{2}$ | (n.u.) | 0.9 ± 0.0 | 0.7 ± 0.0 * | 0.8 ± 0.0 * | 1.0 ± 0.0 * |
| SampEn | (n.u.) | 1.2 ± 0.0 | 1.3 ± 0.0 * | 0.8 ± 0.0 * | 1.2 ± 0.0 * |
| MSE5 | (n.u.) | 1.4 ± 0.0 | 0.9 ± 0.0 * | 0.8 ± 0.0 * | 1.2 ± 0.0 * |
| MSE10 | (n.u.) | 1.4 ± 0.0 | 0.9 ± 0.0 * | 0.8 ± 0.0 * | 1.4 ± 0.0 * |
| MSE15 | (n.u.) | 1.4 ± 0.0 | 0.8 ± 0.0 * | 0.9 ± 0.0 * | 1.4 ± 0.0 * |
| MSE20 | (n.u.) | 1.4 ± 0.0 | 0.8 ± 0.0 * | 1.0 ± 0.0 * | 1.4 ± 0.0 * |

Table S2: Heart rate variability (HRV) analysis results for the PhysioNet human datasets: healthy young (NSR), atrial fibrillation (AF), congestive heart failure (CHF) and healthy aged (AGING). See main text for definition of each metric and analysis parameters. Reported frequency domain metrics are based on autoregressive (AR) modeling. Results reported as mean ± standard error (SE); $N$ is the number of segments analyzed in each dataset; an asterisk denotes $p<0.01$ in unpaired t-test with the NSR case.
